# A structural, genetic and clinical comparison of CAR-T cells and CAR-NK cells: companions or competitors?

**DOI:** 10.3389/fimmu.2024.1459818

**Published:** 2024-10-04

**Authors:** Alain E. Andrea, Andrada Chiron, Guillaume Sarrabayrouse, Stéphanie Bessoles, Salima Hacein-Bey-Abina

**Affiliations:** ^1^ Department of Biology, Faculty of Arts and Sciences, Saint George University of Beirut, Beirut, Lebanon; ^2^ Université Paris Cité, Centre National de la Recherche Scientifique, Institut National de la Santé et de la Recherche Médicale, Unité des Technologies Chimiques et Biologiques pour la Santé (UTCBS), Paris, France; ^3^ Clinical Immunology Laboratory, Groupe Hospitalier Universitaire Paris Saclay, Hôpital Bicêtre, Assistance Publique-Hôpitaux de Paris, Le-Kremlin-Bicêtre, France

**Keywords:** CAR-T cells, CAR-NK cells, Cytokines, Hematological malignancies, Off-the-shelf Immunotherapies

## Abstract

In recent years, following the groundbreaking achievements of chimeric antigen receptor (CAR) T cell therapy in hematological cancers, and advancements in cell engineering technologies, the exploration of other immune cells has garnered significant attention. CAR-Therapy extended beyond T cells to include CAR natural killer (NK) cells and CAR-macrophages, which are firmly established in the clinical trial landscape. Less conventional immune cells are also making their way into the scene, such as CAR mucosal-associated invariant T (MAIT) cells. This progress is advancing precision medicine and facilitating the development of ready-to-use biological treatments. However, in view of the unique features of natural killer cells, adoptive NK cell immunotherapy has emerged as a universal, allogenic, “off-the shelf” therapeutic strategy. CAR-NK cytotoxic cells present targeted tumor specificity but seem to be devoid of the side effects associated with CAR-T cells. CAR-NK cells appear to be potentially promising candidates for cancer immunotherapy. However, their application is hindered by significant challenges, particularly the limited persistence of CAR-NK cells in the body, which poses a hurdle to their sustained effectiveness in treating cancer. Based upon the foregoing, this review discusses the current status and applications of both CAR-T cells and CAR-NK cells in hematological cancers, and provides a comparative analysis of the structure, genetics, and clinical outcomes between these two types of genetically modified immune cells.

## 1 Introduction

Cellular immunotherapy [also known as adoptive cell therapy (ACT)] has produced significant progress in the fight against cancer by enabling physicians to harness the power of engineered immune cells (1). A highly promising anticancer ACT strategy involves the incorporation of chimeric antigen receptors (CARs) on T lymphocytes or (more recently) natural killer (NK) cells or macrophages (2, 3). CAR-modified immune cells (whether either autologous or allogenic, i.e. purified from patients or donors, respectively) are designed to specifically target tumor cells and have proven to be accurate and efficacious in the treatment of cancer. CAR-T cell immunotherapy has been evaluated in thousands of clinical trials, many of which are ongoing (4). However, CAR-T cell therapy faces various challenges, including (i) an elevated risk of adverse events, such as cytokine release syndrome (CRS), immune-effector-cell-associated neurotoxicity syndrome (ICANS), and on-target off-tumor effects (i.e. reactions against antigens expressed in healthy tissues), and (ii) a lack of efficacy and thus disease relapse related to tumor antigen escape. Moreover, allogeneic CAR-T cell therapy faces additional hurdles, such as the need for human leukocyte antigen (HLA) matching to prevent life-threatening graft-versus-host disease (GvHD) (5).

Furthermore, CAR-T cell therapy is confronted with manufacturing problems because of the limited T cell sources (5). Extensive research efforts to overcome these challenges are ongoing, and various experts have reviewed the literature on these hurdles in the context of both hematological and solid cancers (5, 6). Alternative sources of immune cells for CAR immunotherapy are being actively evaluated, and interest in NK cells is growing because of the latter’s unique characteristics (7).

Here, we review, compare and contrast the structural (i.e. engineering-related), genetic and clinical characteristics of CAR-T cells vs. CAR-NK cells, from the bench to the bedside.

## 2 CAR structure

### 2.1 Different generations of CAR-T cells and CAR-NK cells

#### 2.1.1 CAR-T cell engineering

CD4+ and CD8+ T cells have a crucial role in tumor immunosurveillance via the recognition of tumor-associated antigens (TAAs), which results in the secretion of various cytokines and chemokines and then a powerful cytotoxic response against cancer cells (8, 9). However, tumors can hijack the body’s immune defenses via various escape mechanisms, such as the downregulation of major histocompatibility complex (MHC)-I expression (10), the restriction of antigen recognition (11), the accentuation of immune-checkpoint-mediated inhibitory signaling (leading to immune dysfunction or exhaustion) (12), and the creation of an immunosuppressive tumor microenvironment (TME) (13). Therefore, T cells genetically engineered to express a synthetic CAR were developed so that immune cells could be reprogrammed to specifically target cancer cells and overcome some of these hurdles. One of the main advantages of CAR-T cells is their ability to recognize tumor-expressed TAAs and eliminate the target cell in an MHC-independent manner. Recent CAR-T cell engineering strategies can also potentiate tumor infiltration, counteract the immunosuppressive TME, and overcome inhibition by negative immune checkpoint receptors (6).

CAR-T cell therapy is mainly based on the infusion of genetically engineered autologous T cells. Allogeneic CAR-T cells is progressing with regard to “off-the-shelf” availability and scalability; challenges like GvHD and immune rejection need to be addressed and so the current focus is still on well-established autologous methods (14, 15).

**Figure 1:**
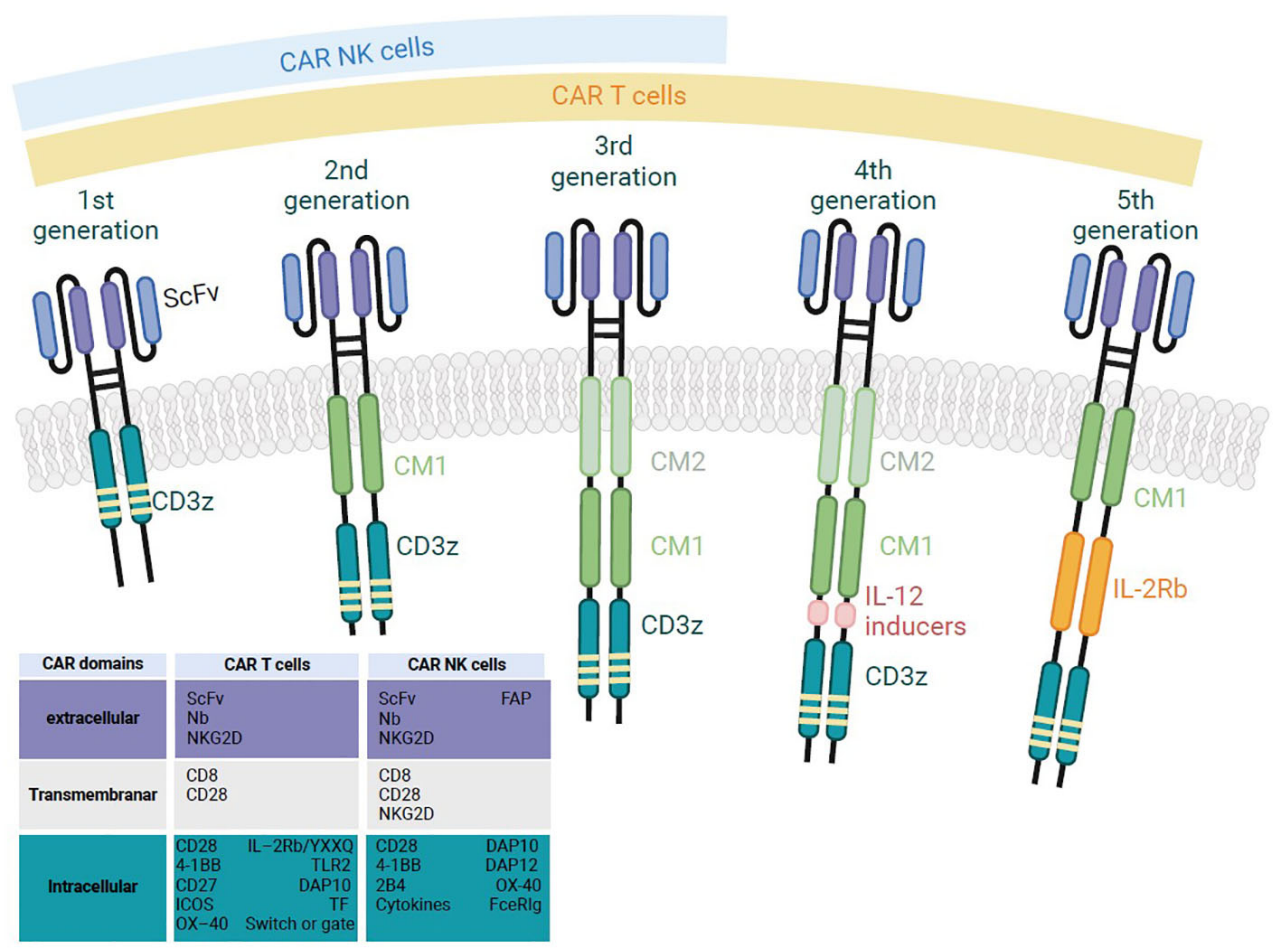
A schematic diagram of the structure of the successive generations of CAR-T and CAR-NK cells. First generation CAR-T/NK cells have only CD3ζ in their signaling domain and lack co-stimulatory molecules. Second generation CAR-T/NK cells include CD3ζ and one co-stimulatory molecule, enabling dual signaling pathways. Third generation CAR-T/NK cells combine CD3ζ with multiple co-stimulatory molecules for enhanced signaling. Fourth generation CAR-T cells, also known as T cells redirected for universal cytokine-mediated killing (TRUCKs), are similar to third generation, but they are a specific type of armored CAR-T cells that produce and secrete cytokines to promote tumor killing. Fifth generation CAR-T cells have an additional intracellular domain compared to earlier generations. These CARs include truncated intracellular domains from cytokine receptors (such as fragments of the IL-2R chain) that feature motifs for binding transcription factors like STAT-3/5.

The CAR combines the specificity afforded by an extracellular antigen-specific recognition domain with the activation afforded by an intracellular signaling domain. Hence, a CAR has four main components: (i) the extracellular, antigen-binding domain consisting of a single-chain variable fragment (scFv) derived from an antibody, (ii) a hinge region derived from CD8, CD28, IgG1 or IgG4 molecules, (iii) a transmembrane domain (TMD) usually derived from type I proteins, such as CD3ζ, CD4, CD8α, or CD28, and (iv) an intracellular signaling domain (SD) containing one or more co-stimulatory domains (CD), such as CD28, 4-1BB (CD137), CD27, MYD88, CD40, OX40 (CD134) or inducible T cell co-stimulator/ICOS (CD278) molecules. CARs can be classified into five generations (**Figure 1**). The first three generations have been extensively described (16), with a focus on the intracellular domains’ impact(s) on the resulting CAR-T cells’ cytotoxic potential and therapeutic efficacy (17).

#### 2.1.2 CAR-NK cell engineering

NK cells are the main cytotoxic innate lymphoid effector cells and are involved in cancer immunosurveillance (18). Clinical studies of patients with various cancers have shown that NK cell activation is associated with better clinical outcomes (18). Unlike T cells, NK cells recognize and kill infected or cancerous cells in an MHC-independent manner. For this reason, NK-based ACT is associated with lower risks of adverse events (19, 20). NK cells kill target cells through finely tuned missing-self, induced-self and antibody-dependent cell-mediated cytotoxicity (ADCC) mechanisms (21, 22). Between 40% and 90% of cancers downregulate their MHC-I expression (23), which enables tumor cells to evade CD8+ T cell-mediated recognition but renders them sensitive to NK cells (24). Activated NK cells are strongly cytotoxic and produce the pro-inflammatory cytokines interferon gamma (IFN-γ) and tumor necrosis factor alpha (TNF-α). Thus, CAR-NK cell therapy has emerged as a prominent area of research and a promising new approach to cancer treatment. Furthermore, the manufacturing techniques related to various sources of NK cells might make this therapeutic strategy a good option for “off-the-shelf” therapy and immediate clinical use; this would avoid the long latency periods inherent to the preparation of autologous therapeutic products (25). Indeed, clinical-grade CAR-NK therapies could be produced from peripheral blood (PB), umbilical cord blood (UCB), NK cell lines (NK-92), and NK cells generated from induced pluripotent stem cells (iPSCs) or CD34^+^ hematopoietic stem cells (HSCs) (19, 26, 27).

As with CAR-T cells, CAR-NK cells can be classified into several generations as a function of how their intracellular domains are organized (**Figure 1**). First-generation CAR-NK cells have a single CD3ζ activation SD, while second- and third-generations include one or two additional CD, respectively. These intracellular domains come from conventional T cell-associated activating receptors (like CD28, ICOS, 4-1BB, CD27 and OX40) or NK-specific signaling molecules (like DAP10, DAP12, DNAM-1 and 2B4); the latter are reportedly more suitable for potentiating NK cell activation (28–32). Several preclinical studies have been conducted to evaluate the potential of the different TMD and intracellular domains, and also the best combinations between these domains in terms of NK cytotoxicity, *in vivo* survival capacity and ultimately antitumor activity. For example, Xu et al. showed that anti-CD5 CAR-NK cells expressing the NK-specific receptor 2B4 displayed greater antitumor activity than anti-CD5 CAR-NK cells expressing 4-1BB, due to greater cytotoxicity *in vitro* and *in vivo* and greater IFN-γ and TNF-α production (30). Moreover, Huang et al. provided a new CAR screening platform that may facilitate CAR-NK design and showed that the *in vitro* cytotoxicity against hepatocellular carcinoma (HCC) cells was greater with DNAM-1-2B4-glypican 3 (GPC3)-targeted CAR-NK cells than with anti-GPC3 CAR-NK cells containing other co-stimulatory domains (CD3ζ, CD28-CD3ζ, DNAM-1-CD3ζ and 2B4-CD3ζ) (33). In fact, concurrent stimulation of CD16 and other activating receptors (namely 2B4, NKG2D, and DNAM-1) led to a greater intracellular Ca^2+^ concentration than activation of CD16 alone (34).

Interestingly, in a recent study published in June 2024, Wang et al. developed seven different CD19 CAR-NK cells and evaluated their antitumor activity and persistence *in vivo*. The results indicated that all CAR constructs improved tumor-killing capacities and prolonged survival in mice with tumors. Notably, CAR1 (CD8 TMD-CD3ζ SD) engineered NK cells demonstrated superior efficacy in treating tumor-bearing mice and showed enhanced persistence when combined with the OX40 CD. In addition, survival rates were notably better in mice treated with CAR1, CAR2 (CD8 TMD-FcϵRIγ SD), CAR3 (CD8 TMD-OX40 CD-CD3ζ SD), and CAR4 (CD8 TMD-OX40 CD- FcϵRIγ SD) NK cells compared to those treated with CAR5 (CD28 TMD-FcϵRIγ SD), CAR6 (CD8 TMD-4-1BB CD-CD3ζ 1-ITAM SD), and CAR7 (CD8 TMD-OX40 CD-CD3ζ 1-ITAM SD) engineered NK cells (35). This comparative study revealed the importance of the nature of TMD, SD and CD, as well as the associations between these domains. It is too early to determine the best CAR design, as studies are still ongoing and no consensus has yet been reached.

### 2.2 Armored CAR-T cells and CAR-NK cells

#### 2.2.1 Generation of armored CAR-T cells and the latest generation of CAR-T cells

Fourth-generation CAR-T cells are also known as T cells redirected for antigen‐unrestricted cytokine‐initiated killing (TRUCKs). From 2011 onwards, researchers have investigated the co-expression of one or more cytokines or the combination of their receptors to generate gene-edited, interleukin (IL)-armored, anticancer immune cells. TRUCKs represent a significant advance in the field of CAR-T cell therapy, and TRUCKs with various ILs (e.g. IL-2, -7, -12, -15, -18, -21 and -23) constitute an active field of research.

Regarding the γc-dependent cytokines, IL-7, IL-15, and IL-21 have been extensively investigated with a view to overcoming the drawbacks of IL-2 and improving the quality of cellular products. IL-15 is known to be a potent immunostimulatory cytokine that modulates innate and adaptive immune responses.

It was described as having the greatest potential in cancer immunotherapy by the US National Cancer Institute in 2008 (36), so that various research groups have explored the addition of IL-15 to CAR-T cell engineering. Hoyos et al. engineered CAR (iC9/CAR19/IL-15 T) T cells with a greater expansion potential (10-fold greater *in vitro* and 3- to 15-fold greater *in vivo*), greater persistence, and stronger antitumor effects in a SCID lymphoma human xenograft model (37). Similarly, Hurton et al. engineered a CAR19 T cells with a membrane-bound IL-15 (mbIL-15) fusion protein (mimicking the unique physiological mechanism of IL-15 trans-presentation) that showed greater activity and a strong memory phenotype in a humanized NOD-SCIDγ mouse model (38).

IL-15 has also been integrated into other CARs to target other types of tumor; these include IL13Ra2-CAR (39) and fibroblast growth factor-inducible 14 (Fn14-CAR) (40) for glioblastoma and GD2-CAR (41) for neuroblastoma. The results of these studies have consistently shown that IL15-armored CAR-T cells exhibit potent antitumor efficacy and enhanced persistence *in vivo*. The first clinical demonstration of the mbIL-15-CAR-T cells’ good levels of safety and efficacy featured a patient with B-cell acute lymphoblastic leukemia (B-ALL) and in whom CD19- and CD22-CAR-T cell therapies had failed (42). The patient received an infusion of CAR19-4-1BB-CD3 ζ -mbIL15 T cells and was achieved a complete response for 5 months, despite a heavy tumor burden. However, the tumor eventually started to progress again following CD19 antigen escape (42).

**Figure 2:**
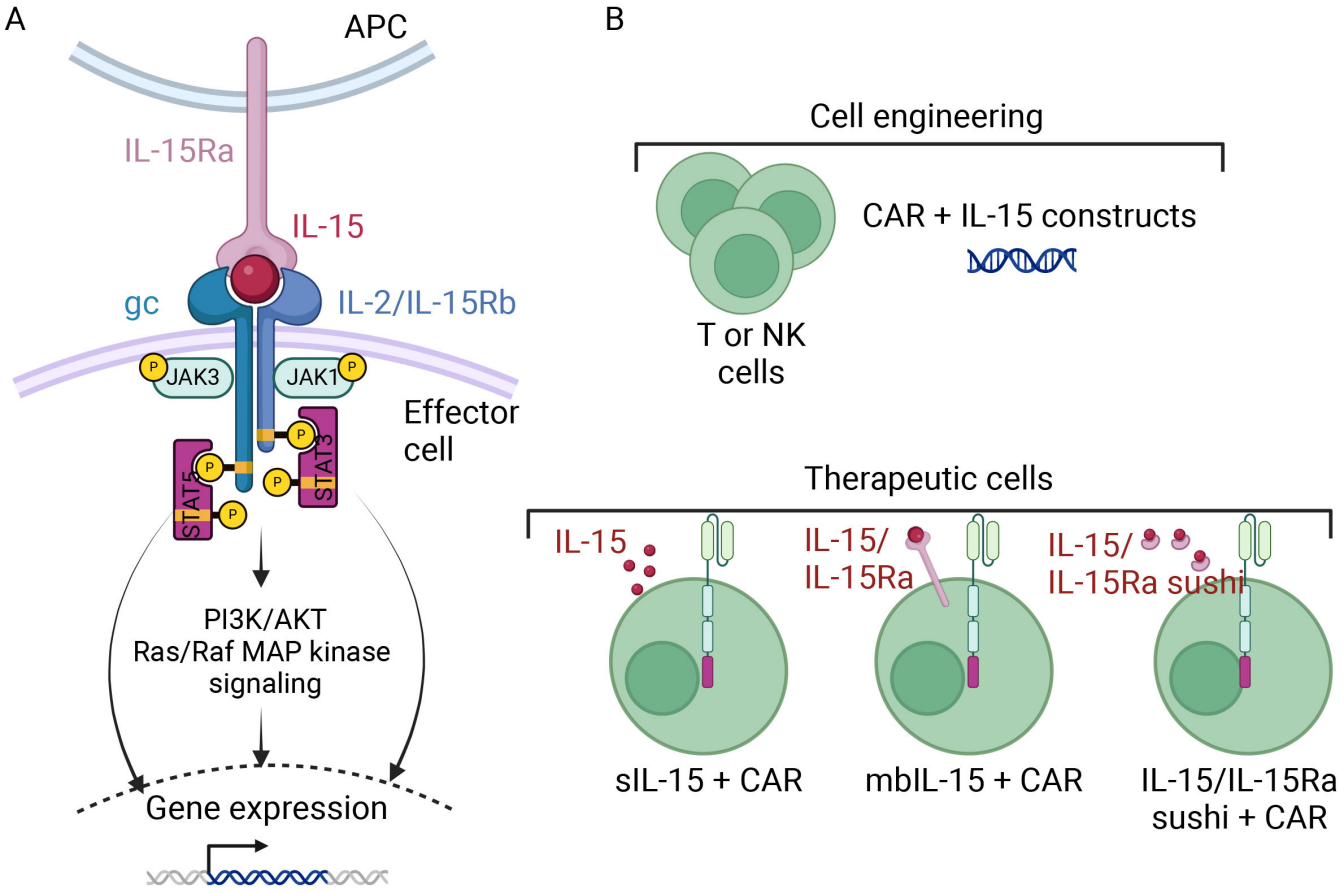
A schematic diagram of CAR-T and CAR-NK cell armoring with IL15 [Adapted from Zhou Y et al., 2022 (86)]. **(A)** The signaling cascade of IL-15 and its receptor complex involve IL-15 being presented on antigen-presenting cells through IL-15Rα, which binds with the β chain (IL-2/15Rβ) and the common γ chain (γc) complex on effector cells. Activation of the β and γc receptors initiates intracellular signaling via the Janus kinase pathway, activating signal transducer and activator of transcription (STAT) proteins downstream. Phosphorylated STATs then translocate to the nucleus, altering gene expression. **(B)** T or NK cells can be armored with IL-15 to serve as therapeutic cells. They can be equipped, beside the CAR, with either secretory IL-15 (sIL-15), membrane-bound IL-15 (mbIL-15), or IL-15, IL-15Ra fusion protein.

The comparative analysis of two Phase I studies (one evaluating GPC3 CAR-T cell therapy (NCT02932956, NCT02905188) and the other evaluating the same CAR combined with IL-15 expression (NCT04377932, NCT05103631) revealed higher peak expansion, a higher response rate and similarly effective tumor trafficking for the IL-15 GPC3 CAR-T cells (Steffin et al. American Society of Pediatric Hematology/Oncology Conference (ASPHO 2023)). It should be also mentioned the trial of IL-15 GD2 CAR-T cell therapy for neuroblastoma and osteosarcoma (NCT03721068), which is underway. Lastly, a Phase I study in T-ALL patients found that the administration of CD5-IL15/IL15sushi CAR led to a rapid reduction in the malignant T cell count within 4 weeks of infusion (43) (see section 4.1.2). These results suggest that the incorporation of IL-15 and its receptor complex might be a safe, valuable means of potentiating CAR-T cell therapy (**Figure 2**).

As seen with IL-15, the overexpression of IL-21 drives long-term T cell persistence *in vivo* (44). Batra et al. designed GPC3 CAR-T cells co-expressing IL-15 and IL-21 and observed robust expansion and sustained persistence of these T cells in their HCC xenograft models; the resulting tumor control and survival rates were higher than those with CAR-T cells equipped with only one cytokine or the other (45). These results provide a strong rationale for evaluating these CAR-T cells in patients with liver cancer (NCT04715191, NCT02932956) (45).

With regard to the cytokines with the most potent inflammatory effects, considerable efforts have been made to establish IL-12 in tumor therapy. The combination of CAR-T cell therapy with constitutive or inducible IL-12 expression has exhibited a remarkable level of efficacy in the treatment of several solid tumors in preclinical models (46–49). However, in a Phase I clinical trial, stable disease was the best observed response to treatment with constitutively IL-12-secreting CAR-T cells (NCT02498912), furthermore, two-thirds of the lymphodepleted patients showed dose-limited toxicity (50). A clinical trial evaluating the efficacy and safety of inducible IL-12 CAR-T cell therapy is ongoing (NCT03542799) (49).

CAR-T cells that secrete IL-18A might constitute a safer alternative to TRUCK IL-12 cells. IL-18 induces IFN-γ expression in T cells and has been shown to activate monocytes and lymphocytes without causing severe toxicity in clinical trials (51). Forced expression of other cytokines (such as IL-23 and granulocyte macrophage colony-stimulating factor) also increases the persistence and antitumor effects of CAR-T cells (52, 53), and other cytokines are now being investigated [for reviews, see (48, 49)].

The fifth and latest generation of CAR-T cells involves the addition of an intracellular domain for cytokine signaling receptors; for example, the IL-2 receptor β-chain (IL-2Rβ) activates the Janus kinase/signal transducers and activators of transcription (JAK-STAT) signaling pathway in an antigen-specific manner. This modification makes it possible to introduce the third signal required physiologically for T cell activation and proliferation (**Figure 1**) (48). This activation promotes the proliferation and persistence of CAR-T cells and enhances their *in vivo* antitumor effects (54, 55).

#### 2.2.2 Generation of armored CAR-NK cells

The fourth generation of armored CAR-NK cells has been engineered to express both cytokines and contain co-stimulatory domains, in order to mitigate the cells’ short lifespan *in vivo*. As performed with CAR-T cells, researchers have engineered armored NK cells that produce cytokines ectopically; this approach increases the persistence and proliferation of CAR-NK cells through the autocrine production of essential cytokines (56–60). Almost every aspect of NK cell immunity is regulated by IL-15, and so the value of this cytokine or its analogs in the treatment of various cancers are being investigated in several ongoing clinical trials (61). NK cells have been genetically modified to produce secretory IL-15 (sIL-15) or mbIL-15 and then evaluated in several preclinical and clinical models of acute myeloid leukemia (AML) (62–64), lymphoma (65), pancreatic cancer (66), and multiple myeloma (MM) (64); the NK cell survival rates and levels of antitumor efficacy were significantly improved (**Figure 2**).

The most encouraging results came from the Phase I/II clinical trial conducted by Rezvani’s group, UCB-derived CD19 CAR-NK cells engineered to express sIL-15 were reportedly safe in the treatment of 37 patients with relapsed or refractory (r/r) CD19-positive malignancies (NCT03056339) (20, 67) (see section 4.2.1).

It should nevertheless be noted that CAR-NK cells engineered to secrete IL-15 caused early death in an immunodeficient mouse model engrafted with human MV-4–11 AML cells (62). In this recent study, the serum IL-15 concentration rose to more than 1000 pg/ml, which is much higher than the values observed in other studies (57, 66). Further optimization of the constructs might safely regulate the secretion of IL-15 and a clinical trial of NK cells genetically engineered to secrete low concentrations of IL-15 was recently initiated in patients with r/r non-small cell lung cancer (NCT05334329).

Another means of enhancing the activity of genetically modified sIL-15 NK cells involves targeting the down-regulators (i.e. checkpoints) of IL-15 signaling. A recent study in a mouse xenograft model of human Raji lymphoma found that a combination strategy – i.e. the engineering of CAR19 UCB-NK cells to express IL-15, together with disruption of the cytokine-inducible Src homology 2–containing protein (CISH) locus – enhanced the cells’ antitumor cytotoxic activity (65). Other studies have shown that CISH knock-out (KO) in CAR-NK cells derived from iPSCs (68, 69), NK-92 cells or primary NK cells (70) led to greater antitumor activity. The engineering of CAR-NK cells with mbIL-15 is also being explored (71, 72). Similarly, CAR-NK cells expressing IL-15 tethered to the IL15 receptor α-chain (IL15-IL15Rα) showed greater persistence and greater antitumor activity in an ALL xenograft model (73). This strategy has already been included in iPSC-derived CAR-NK cells targeting B-cell maturation antigen (BCMA) in MM (FT576 NK cells) (74, 75) or targeting CD19 in B-cell malignancies (FT596 NK cells) (Fate Therapeutics) (76). These products have been evaluated in clinical trials (NCT04245722, NCT05934097 and NCT04555811).

Even though the use of IL-15-armored CAR-NK cells is promising, there are many unsolved or controversial issues with regard to IL-15 and its pleiotropic effects. Firstly, human IL-15 has at least four functional forms *in vivo*, (i) soluble monomeric IL-15, (ii) the soluble IL-15/IL-15Rα complex, (iii) trans-presented IL-15, and (iv) mbIL-15 (77–79). It is not clear which forms of IL-15 are most abundant under physiological and pathological conditions or which form is best for NK cell expansion *in vivo* (80). Secondly, long-term or repeated exposure to IL-15 results in NK cell hypo-responsiveness, which impairs the cells’ survival, activation, cytotoxicity, and antitumor activity (81, 82). Thirdly, and although IL-15 promotes the antitumor immunity mediated by NK cells and CD8+ T cells, certain tumor-promoting properties of IL-15 and/or IL-15/IL-15Rα have been noted in patients with leukemia or solid tumors (83, 84). Recently, newly designed IL-21-armored CAR-NK cells were found to show significantly greater IFN-γ and TNF-α production and greater degranulation than IL-15-armored counterparts; this resulted in greater activity of CD19-CAR-NK cells against CD19-positive lymphoma *in vitro* (85).

## 3 Gene transfer strategies

### 3.1 Viral vector-based gene transfer

The viral vectors employed in CAR-T cell production are derived either from integrating γ-retroviral (RV) or lentiviral vectors (LV), which enable prolonged, stable transgene expression (87). Both types of viral vector have some drawbacks, including limited insert size, difficulty in generating high titers of stable vector particles, and the risk of insertional mutagenesis. Fortunately, this risk has virtually disappeared following the introduction of self-inactivating (SIN) viral vectors (88). Of the six CAR-T cell products approved by the FDA since 2017, two (Yescarta^®^ and Tecartus^®^) use RV and four (Kymriah^®^, Breyanzi^®^, Abecma^®^, and Carvykti^®^) use the third-generation SIN-LV (88, 89).

**Figure 3:**
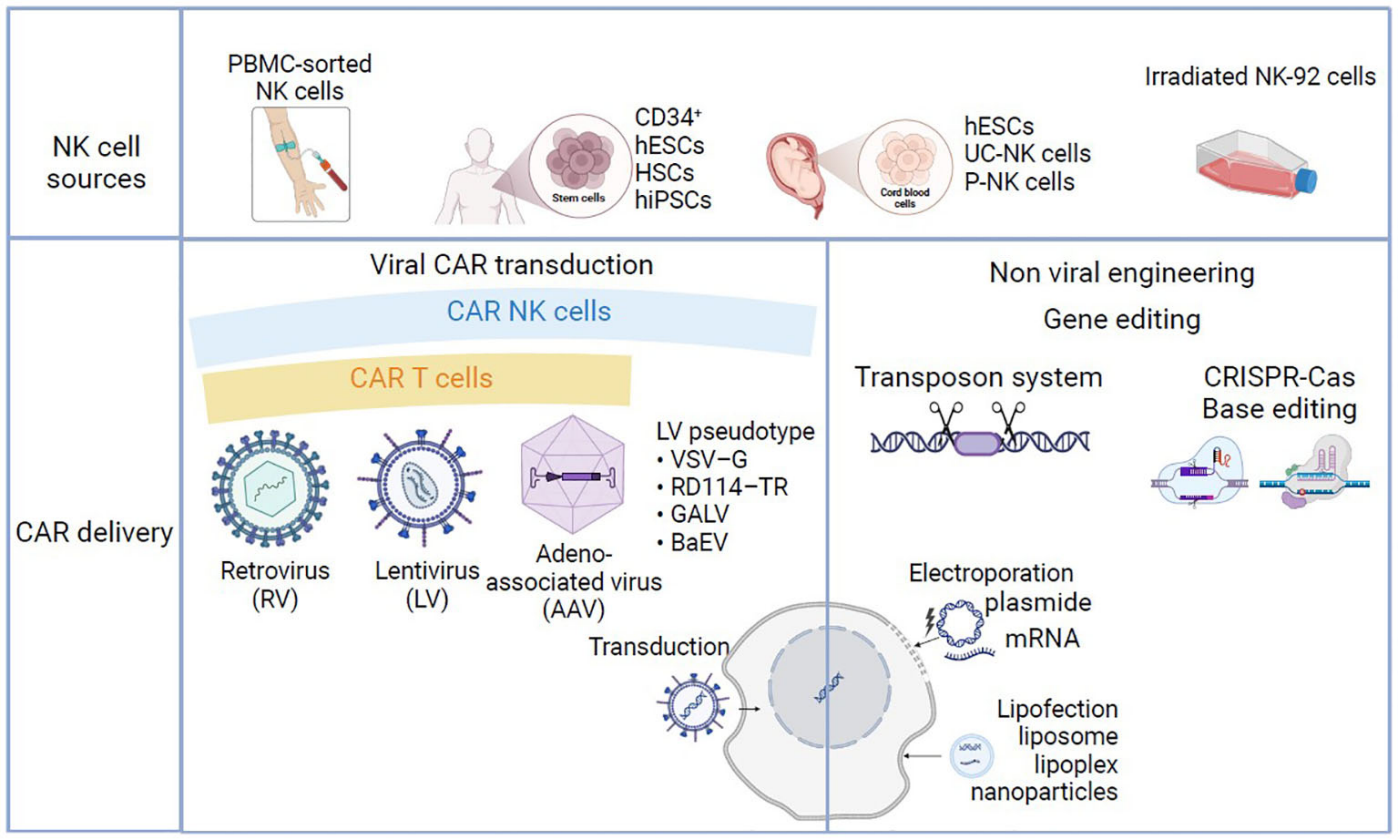
Manufacturing processes in CAR-NK and CAR-T-cell-based therapy. NK cells can be obtained from peripheral blood (PB-NK), CD34-positive hematopoietic stem cells (HSCs), umbilical cord blood (UCB-NK) induced hematopoietic stem cells (iPSCs), or *in vitro* cultured cell lines (NK-92). T cells can be obtained from peripheral blood apheresis (not shown). T and NK cells can be modified to express specific CAR using viral transduction with γ-retrovirus, lentivirus or adeno-associated virus, or non-viral strategies such as transposon system, CRISPR-Cas base editing. Non-viral gene transfer/delivery techniques are based on lipofection or electroporation/nucleofection.

Compared with other hematopoietic cells in general and T cells in particular, viral gene delivery to primary NK cells has proven to be less effective (90). The innate immune properties of NK cells probably contribute to the low viral transduction efficiency and ultimately trigger apoptosis of these cells (91). This phenomenon might be due (at least in part) to the greater expression and then subsequent activation of pattern recognition receptors that detect foreign genomic material as pathogen-associated molecular patterns (92). However, some research groups have achieved good yields with viral transductions (93) (**Figure 3**). LV transduction has been notably improved by the introduction of novel enhancers that aid viral entry (94) or inhibit antiviral cellular signaling (95). Furthermore, Polten et al. recently used SIN alpha-RV to genetically modify NK cells with a third-generation CAR, with a view to specifically targeting and eliminating cervical cancer cells. This approach used anti-fibroblast activation protein and anti-mesothelin (MSLN) CAR-NK cells and holds promise for clinical applications. The study’s results (Abstract at the European Society of Gene and Cell Therapy’s 30^th^ Annual Congress) not only pave the way for potential treatments for cervical cancer but also indicate that the approach can be extended to other gynecological malignancies and even to fibrotic diseases.

### 3.2 Non-viral strategies for gene transfer

Non-viral techniques for gene transfer have recently emerged as potentially safer and less expensive than viral vectors and are now being evaluated for use in CAR cell design (**Figure 3**). Furthermore, the remarkable development of CRISPR-Cas9 and transposon technologies has enabled transgene integration in the absence of genome-integrating viral vectors. The initial strategy for CAR-cell engineering through non-viral gene delivery techniques was based on electroporation/nucleofection. The electroporation studies carried out in the early 2000s highlighted the high degree of heterogeneity in transfection efficiency and cell viability (96, 97). These parameters have been improved with the new developed protocols and electroporation is still a valuable approach for *ex vivo* T and NK cell engineering until the other non -viral methods have taken root for good (98, 99).

Alternatively, to viral- and electrical mechanical-based gene delivery strategies, nanocarriers have been investigated owing to their low cytotoxicity, low cost, and ease of use. To date, polymers and lipids are the most commonly used biomaterials in CAR cell development (100, 101). Their use in pre- clinical applications of CAR-T cell engineering has been explored with success. Nevertheless, in most pre-clinical studies, the transfection values obtained in cell lines are substantially higher than those obtained in primary cultures (101). In a recent study by Golubovskaya et al., mRNA-lipids nanoparticles (LNP) technology was used to transfect NK cells derived from primary PBMCs. The encapsulation of CD19-CAR and BCMA-CAR mRNA within LNPs resulted in remarkable CAR expression levels in NK cells (78% and 95% respectively) and significantly enhanced cytotoxic activity (102).

With regard to the tools used to integrate the transgene, transposon systems (notably Sleeping Beauty (SB) and PiggyBac (PB)) have received significant attention for the cost-effective gene delivery into human lymphocytes in general and the generation of CAR-T cells in particular (103, 104). The objective is to make CAR-therapy more affordable and more widely available to patients (105, 106). When compared with RV and LV vectors, transposon systems offer a higher level of safety because they do not integrate into transcriptionally active genes (i.e. near transcription start sites) or genes outside transcriptional units but which are involved in cancer.

Many researchers have used transposon systems to generate primary CAR-T cells (104). In a study conducted by Yang et al, a CD133 CAR-T cell secreting PD-1 blocking scFv was built by using an SB transposon system and minicircle technology. The CD133 CAR-T cells demonstrated significant antitumor activity against HCC, both *in vitro* and in mouse xenograft models (107). Singh et al. conducted a Phase I clinical trial (NCT02807883) evaluating the safety of CD19 CAR-T cells transduced with an SB transposon system, in 14 patients with B-cell malignancies. They reported that their SB-based CAR-T cell therapy is safe, cost-effective and shows encouraging antitumor activity (108).

Regarding NK cell engineering, encouraging results have been achieved in different preclinical studies using SB or PB transposon system to introduce, (i) a CD33-CAR into cytokine-induced killer (CIK) cells in an AML xenograft model (109), (ii) a MSLN-CAR into NK-92MI cells in a pancreatic cancer model (110), (iii) a CAR-NK cells expressing both NKG2D CAR and IL-15 in an AML xenograft model (63). An innovative approach for CAR-NK cell engineering has recently employed the SB transposon/transposase-based system and a DNA minicircle. In this strategy, Bexte et al. used the SB100X mRNA, rather than the protein and showed better outcomes in terms of gene transfer stability and cell toxicity. Their SB-transposed cells’ achieved strong antileukemic potential both *in vitro* and *in vivo* in a mouse ALL xenograft model (111).

In recent years, many research groups have investigated the use of CRISPR technology to improve the preclinical performance of T and NK cell immunotherapies. The first successful clinical application of CRISPR-engineered TCR-T cells was reported in 2020 (112). CRISPR-Cas9 can be used for the multiplex KO of inhibitory molecules or receptors, in order to enhance CAR-T cell expansion and persistence in the treatment of both hematological cancers and solid tumors. For instance, studies employing CRISPR-Cas9 to disrupt PD1 expression in CAR-T cells have shown enhanced cytotoxicity and reduced exhaustion in glioblastoma with anti-EGFRvIII CAR-T cells (113), increased cytokine production, improved tumor control, and relapse prevention in breast carcinoma with anti-MSLN CAR-T cells (114), and increased *in vivo* antitumor activity against HCC with anti-GPC3 CAR-T cells (115).

Similarly, specific gene editing strategies for primary NK cells or (to a lesser extent) CAR-NK cells have targeted immune checkpoint receptors (PD1 and TIM3), NK inhibitory receptors (the siglec-7 receptor and NKG2A), CISH, and NK activation regulators (e.g. a disintegrin and metalloproteinase (ADAM)-17) (116). For example, targeting the PD1/PDL1 axis via PD-1 KO and ADAM17 KO has been shown to increase antitumor activity (i) in a xenograft model of ovarian cancer and (ii) against chronic myeloid leukemia (CML) and AML cell lines *in vitro* (117).

The main features (advantages and disadvantages) of CAR-T cells and CAR-NK cells are summarized in the **Table 1** below.

**Table 1:** Main characteristics of CAR-T and CAR-NK cells.

Parameters	CAR-T cells	CAR-NK cells
Sources	Patient’s autologous T cells from PB apheresis (with some attempts made using allogeneic cells)	Various sources: PB, UCB, NK-92 cell line, iPSCs or CD34+ HSCs
Risk of GvHD	High risk with allogenic source	Very low risk with allogenic source which implies that it can be off-the-shelf product.
CRS	High risk	Low risk
Tumor Targeting	MHC-independent function	MHC-independent function
Transduction	Typically done using viral vectors with high efficiency and established protocols. Cost-effective non-viral methods are being explored	More challenging; lower transduction efficiency with viral vectors. Alternative non-viral methods (electroporation, LNPs) are being explored to improve efficiency and cost-effectiveness.
Expansion *in vitro*	Robust expansion protocols established, but time-consuming and requires specific cytokines	Generally, more challenging; NK cells have lower expansion rates and also require cytokines (+/-feeder cells)
Persistence *in vivo*	Long-term persistence	Short persistence
Clinical Efficacy	Highly effective against certain hematological cancers	Promising results, especially in hematological cancers
Regulatory Status	Six FDA-approved products	Still in early stages of clinical use

PB, peripheral blood; UCB, umbilical cord blood; iPSC, induced pluripotent stem cell; HSC, hematopoietic stem cell; GvHD, graft versus host disease; CRS, cytokine release syndrome; MHC, major histocompatibility complex; LNP, lipid nanoparticles.

## 4 A comparative analysis of the clinical efficacy of CAR-T cells and CAR-NK cells in the treatment of hematological cancers

### 4.1 CAR-T cells in hematological cancer

Since clinical trials of CAR-NK cell therapies are relatively rare, compared with the large number of clinical trials of CAR-T cells, we shall solely focus on a comparison of the two types of immunotherapy in the field of hematological cancers and the clinical trials mentioned below refer to data collected up to April 30^th^, 2024. As mentioned above, the FDA has approved six CAR-T products (**Table 2**).

**Table 2:** Overview of CAR-T cell therapies approved by the FDA for the treatment of B-cell malignancies.

Generic name	Brand name	CAR	Hinge, TM/ signaling domains	First indication	Pivotal trial(s)	First FDA approval	Reference
Tisagenlecleucel	Kymriah^®^	CD19	CD8α,CD8α/ 4-1BB.CD3ζ	r/r B-ALL	ELIANA (NCT02435849) ENSIGN (NCT02228096)	Aug. 30^th^, 2017	(118)
Axicabtagene ciloleucel	Yescarta^®^	CD19	CD28,CD28/ CD28.CD3ζ	r/r LBCL	ZUMA-1 (NCT02348216)	Oct. 18^th^, 2017	(119)
Brexucabtagene autoleucel	Tecartus^®^	CD19	CD28,CD28/ CD28.CD3ζ	r/r Mantle Cell Lymphoma	ZUMA-2 NCT02601313	Jul. 24^th^, 2020	(120)
Lisocabtagene maraleucel	Breyanzi^®^	CD19	IgG4,CD28/ 4-1BB.CD3ζ	r/r LBCL	TRANSCEND-NHL-001 (NCT02631044)	Feb. 5^th^, 2021	(121)
Idecabtagene vicleucel	Abecma^®^	BCMA	CD8α,CD8α/ 4-1BB.CD3ζ	r/r MM	KarMMa (NCT03361748)	Mar. 26^th^, 2021	(122)
Ciltacabtagene autoleucel	Carvykti^®^	BCMA	CD8α,CD8α/ 4-1BB.CD3ζ	r/r MM	CARTITUDE-1 (NCT03548207)	Feb. 28^th^, 2022	(123)

ALL, acute lymphocytic leukemia; BCMA, B-cell maturation antigen; CAR, chimeric antigen receptor; LBCL, large B-cell lymphoma; MM, multiple myeloma; r/r, relapsed or refractory; TM, transmembrane.

#### 4.1.1 B-cell lymphoblastic leukemia/lymphoma

Clinical trials of CD19 CAR-T cell therapy have yielded promising outcomes for patients with unfavorable prognoses. By conducting a simple search on ClinicalTrials.gov, we note that there are over 400 clinical trials involving CD19 CAR-T cells in cancer treatment. Since clinical trials targeting CD19 have been extensively described in the literature (124), we will avoid detailing them in this article. However, many ongoing clinical trials of CAR-T cell therapy are targeting different markers with the aim of achieving even better outcomes in B-cell malignancies, particularly in patients who have relapsed after receiving CD19-targeting CAR-T cells due to CD19 downregulation or loss.

Pan et al. conducted a study (ChiCTR-OIC-17013523) of 34 children and adults with r/r B-ALL and who had not responded to anti-CD19 CAR-T cell therapy. After receiving anti-CD22 CAR-T cells, 80% of the evaluated patients (24 out of 30) achieved either a complete remission or remission with incomplete count recovery by day 30 post-infusion. Of the patients who achieved a CR, 7 did not require further treatment, and 3 of the 7 maintained remission at 6, 6.6 and 14 months post-infusion (125). Spiegel et al. conducted a Phase I clinical trial (NCT03233854) of a bispecific CAR (targeting both CD19 and CD22) in adults with r/r B-ALL and large B-cell lymphoma (LBCL). For the 17 patients with B-ALL, the response rate was 100% and the complete response (CR) rate was 88%. For the 21 patients with LBCL, the response rate was 62% and the CR rate was 29%. The relapse rate was 50% among the B-ALL patients (5 out of 10) and 29% among the LBCL patients (4 out of 14); the relapses were characterized by low or null CD19 expression and did not appear to be linked to CD22 expression levels (126). CD22 has been the subject of even greater attention in many recent clinical studies of CAR-T cells, both as a single target (127–132) and in combination with CD19 (133–140) (for a meta-analysis, see Fergusson et al. (141)).

CD20 has also been targeted in patients with B-cell malignancies. The presence and expression levels of CD20 vary from one B-cell malignancy to another: the lowest levels of CD20 expression are observed in chronic lymphocytic leukemia (CLL) and the highest levels are observed in diffuse large B cell lymphoma (DLBCL) and hairy cell leukemia (142). In 2012, Till et al. initiated the first clinical trial (NCT00621452) of anti-CD20 CAR-T cells in patients with relapsed indolent B-cell and mantle cell lymphoma: the objective response rate (ORR) was 83%, and the CR rate was 50% (143). An early Phase IIa study (NCT01735604) of anti-CD20 CAR-T cells conducted by Zhang et al. subsequently found a favorable response in advanced B-NHL: the CR rate was 54.5% (6 out of 11), the partial response (PR) rate was 27.3% (3 out of 11), and thus the ORR was 81.8%. The remaining two patients showed stable disease (SD). The median progression-free survival (PFS) time was 6 months, and these data were obtained after a median follow-up period of 8 months (144).

In a recent Phase I dose escalation and expansion trial (NCT03019055), Shah et al. tested the efficacy of bispecific anti-CD19/CD20 CAR-T cells in 21 adult patients diagnosed with B-cell NHL or CLL. By day 28, the ORR was 82%, the CR rate was 64%, and the PR rate was 18% (145). In a Phase I clinical trial (NCT04007029) published in March 2023, Larson et al. assessed the safety of bispecific anti-CD19/CD20 CAR-T cells in 10 patients with r/r NHL. Nine of the patients exhibited an objective response, giving an ORR of 90% and a CR rate of 70%. There was one case of relapse after 18 months with a CR; although this patient subsequently achieved full remission after receiving a second dose of anti-CD19/CD20 CAR-T cells. The median PFS time was 18 months, and the median overall survival (OS) time had not been reached by the end of the analysis (a median follow-up period of 17 months) (146).

Additional antigen targets are currently under investigation in both preclinical and clinical studies; for example, receptor tyrosine kinase-like orphan receptor 1 is currently undergoing clinical evaluation (NCT02194374, NCT05588440, NCT05694364 and NCT02706392) (147, 148), and also CD123 (149–152).

#### 4.1.2 T cell lymphoblastic leukemia/lymphoma

CAR-T cell therapy faces significant challenges when applied to the treatment of T cell malignancies. This difficulty arises from the co-expression of many targets on both normal and malignant T cells (153). CD7 is acknowledged to be a pivotal antigen in the treatment of T cell acute lymphoblastic leukemia (T-ALL) and T-lymphoma, primarily because of its widespread distribution on tumor cells (154). However, CD7 is also expressed on normal T lymphocytes, NK cells, and in early-stage of lymphocyte differentiation (155). Consequently, the infusion of anti-CD7 CAR-T cells into patients may unintentionally deplete T and NK cells and thereby increase the risk of opportunistic infections. Furthermore, the uninhibited expression of CD7 by anti-CD7 CAR-T cells might lead to a fratricidal phenomenon. In order to mitigate the latter possibility, researchers are using gene editing techniques to effectively KO the CD7 gene in genetically modified T cells (156).

In a Phase I clinical trial conducted by Pan et al., 20 patients diagnosed with r/r T-ALL were treated with donor-derived anti-CD7 CAR-T cells. The results were highly promising, with a remarkable CR rate of 90%. Even after a median follow-up period of 6.3 months, 15 of the 18 (83%) patients who achieved a CR remained in remission, and only one patient experienced a relapse (characterized by the absence of CD7 expression). Despite being allogeneic, the aforementioned CAR-T cells displayed robust proliferation in all patients and persisted in patients without the need for subsequent HSCT (157). In an open-label Phase I clinical trial (NCT04004637), Zhang et al. administered autologous nanobody-derived fratricide-resistant anti-CD7 CAR-T cells to patients with r/r CD7-positive T- ALL/T-LBL. Three months after the CAR-T cell infusion, the CR rate was 87.5% (7 out of 8). Notably, one patient with leukemia showed a CR with no minimal residual disease (MRD), while another patient with lymphoma maintained a CR for over 12 months (158).

Lu et al. introduced an innovative therapy for generating naturally selected anti-CD7 CAR-T cells from bulk T cells; the fratricidal problem was mitigated by minimizing the availability of CD7 epitopes. In the initial Phase I clinical trial (NCT04572308), 20 patients diagnosed with r/r T-ALL (n = 14) or T-LBL (n = 6) were treated. Nineteen patients achieved a CR with no MRD in the BM by day 28, while five showed an extramedullary CR. With a median follow-up duration of 142.5 days after the CAR-T infusion, 14 patients subsequently underwent allogeneic HSCT; no relapses were observed up to this point. Of the six patients who did not undergo HSCT, 4 remained with a CR after a median duration of 54 days (159). To date, 45 ClinicalTrials.gov-registered trials have used anti-CD7 CAR-T cell therapy in r/r T-ALL or T-LBL. Ten of these are Phase II clinical trials: NCT05059912, NCT06064903, NCT05454241 NCT05909527, NCT04762485, NCT04033302, NCT04689659, NCT05827835, NCT04984356, and NCT05885464.

CD5 is expressed at high levels in many T cell malignancies (and especially in T-ALL and peripheral T cell lymphomas) and is therefore an attractive target in CAR-T cell therapy. In a Phase I clinical trial (NCT04594135), the safety and efficacy of CD5-IL-15/IL-15sushi CAR-T cells were assessed in a patient suffering from r/r T-LBL with central nervous system (CNS) infiltration. The novelty of these anti-CD5 T cells lies in their ability to produce IL-15 plus sIL-15Rα-sushi. The genetically modified T cells rapidly eliminated CNS lymphoblasts and led to remission of the patient’s lymphoma. The lymphoblast count became undetectable four weeks after the CAR-T cell infusion. Even though normal T cells also possess CD5, the patient’s T cell aplasia was short and transient (43). Other clinical trials (e.g. NCT06316856, NCT05596266, NCT05032599, NCT05487495, NCT03081910, and NCT04767308) are currently assessing the efficacy and safety of other anti-CD5 CAR-T cells for the treatment of T cell malignancies.

Other targets are being investigated in preclinical and clinical trials: CD4 (160, 161) (NCT04162340, NCT04973527, NCT04219319, NCT03829540, and NCT04712864), CD30 (162, 163) (NCT04083495, NCT04526834, NCT03049449, NCT04008394, NCT05208853, NCT04653649, and NCT03602157), T cell receptor beta constant 1 (164, 165) (NCT03590574 and NCT04828174), and CD1a (166) (NCT05745181 and NCT05679895).

#### 4.1.3 Acute myeloid leukemia

AML exhibits significant heterogeneity due to the variable presence of distinct chromosomal abnormalities, gene mutations, and gene fusions. Although researchers now have a better understanding of the immunopathological mechanisms underlying this hematological cancer, a single, highly-specific therapeutic target has proven to be an elusive (167). Several surface proteins (such as CD33, CD123, C-type lectin-like molecule-1 (CLL-1), CD7, CD70, immunoglobulin-like transcript 3 (ILT3), NKG2DL, CD38 and FLT3) are potential targets and have entered clinical trials (168). However, there is a notable degree of concern regarding on-target/off-tumor toxicity and thus the risk of SAE; in fact, most of the surface antigens found on AML blasts are also expressed by mature myeloid cells and HSCs (169).

CD33 is typically present on cancer cells in more than 90% of cases of AML (170). A Phase I clinical trial (NCT03126864) with CD33-targeted CAR-T cells enrolled 10 adults with r/r AML but the anti-CD33 CAR-T cells could be infused to only three of them. The patients exhibited leukopenia and two of them had circulating blasts. Two patients experienced CRS, none achieved the clinical endpoints, and all three succumbed to disease progression (171).

Other clinical trials are now assessing the efficacy and safety of CAR-T cells targeting CD33 alone (NCT06326021, NCT05445765, NCT02958397, NCT05473221, NCT05672147, NCT03126864, NCT02799680, NCT04835519, NCT01864902, NCT05984199, NCT03927261, NCT03971799, NCT05105152, and NCT05945849) or in combination with other targets, such as CLL-1 (NCT05467254, NCT05943314, NCT05248685, NCT04010877, NCT05016063, and NCT03795779), CD123 (NCT04156256, NCT03222674, NCT05995041). Moreover, the clinical trial NCT03222674 is distinguished by the evaluation of the combination of different targets such CD38, CD123, CD56, MucI, and CLL-1. CD123 expression is reportedly correlated with a greater risk of treatment failure (172). However, CD123 is emerging as a very promising therapeutic target because of its robust presence in AML and its relatively weak expression by HSCs (172). Anti-CD123 CAR-T cells reduced leukemia *in vivo* while inflicting minimal damage on normal HSCs (173). In January 2020, a clinical trial (NCT04230265) tested UniCAR-T-CD123 cells in r/r AML patients with ≥20% CD123+ blasts. The treatment combined UniCAR-T cells and the recombinant antibody derivative called CD123 target module (TM123), with TM123 given daily for 25 days and UniCAR-T cells on day 1. Out of eight patients, two were excluded and two died before treatment. Of the three who completed it, one had a PR, and two achieved CRi. One CRi patient relapsed after a month, leading to a second TM123 treatment (174).

CLL-1 has also been identified as a valuable target, since it is found on 92% of AML cells but is absent on granulocyte-macrophage progenitors. Importantly, CLL-1 is also present on leukemic stem cells (175). Zhang et al. designed fourth-generation anti-CLL-1 CAR-T cells and administered them to a 10-year-old patient with secondary AML (NCT00846703) after lymphodepleting chemotherapy. The patient achieved a CR with no MRD; however, the CLL-1+ cells were not entirely eradicated until six months after the infusion of CAR-T cells. Nevertheless, a single dose of anti-CLL-1 CAR-T cells led to 10 months of remission in this patient (176). Four children diagnosed with r/r AML were included in a Phase I/II clinical trial (NCT03222674, also conducted by Zhang et al.) of anti-CLL1 CAR-T cell therapy. Three of these patients attained a CR with no MRD. The fourth patient survived for a period of 5 months. Importantly, all of these individuals encountered only mild, controllable adverse events during the course of treatment (177). Three other recent clinical trials (ChiCTR2000041054, NCT04884984, and NCT03222674) have yielded favorable outcomes - suggesting that CLL-1-specific CAR-T cells hold promise in AML therapy (178–180).

#### 4.1.4 Hodgkin lymphoma

The development of the anti-CD30 antibody-drug conjugate brentuximab vedotin created an opportunity to consider CD30 as a target for CAR-Therapy for r/r HL. Advantageously, CD30 is highly prevalent on Hodgkin and Reed-Sternberg cancer cells but not on other cells and tissues, which reduces the risk of adverse events. Wang et al. pioneered the demonstration of the feasibility and safety of anti-CD30 CAR-T cell treatment for r/r HL in a Phase I trial (NCT02259556) involving 18 patients, most of whom had a significant treatment history or multiple tumors (181). The infusion of anti-CD30 CAR-T cells was well tolerated, and only two of the 18 patients experienced SAE (grade ≥3). Of the 18 patients, seven achieved a PR and six showed SD (ORR: 39%) (181). Ramos et al. conducted another Phase I trial (NCT01316146) in nine patients with r/r HL or anaplastic large cell lymphoma, the results of which further underscored the direct impact and safety of anti-CD30 CAR-T cells. A secondary analysis of the endpoints showed an ORR of 33%, including long-lasting responses. Of the seven patients with r/r HL, one achieved a CR that lasted over 2.5 years following a second infusion of anti-CD30 CAR-T cells, another maintained a continuous CR for nearly 2 years, and three experienced temporary SD (182).

Ramos et al. simultaneously conducted two Phase I/II trials (NCT02690545 and NCT02917083), each at a different medical center. The trials involved individuals with r/r HL and the administration of anti-CD30 CAR-T cells after lymphodepletion. The studies’ primary focus was safety, and it is noteworthy that no dose-limiting toxicity was observed - even at the highest treatment dose. CRS was limited to grade 1 and did not require medical intervention. Regarding the treatment’s efficacy, the ORR among the 32 patients with active disease was 72%, and the CR rate was 59%. Among patients with measurable disease, the one-year PFS rate was 41% for individuals having undergone fludarabine lymphodepletion and 61% for individuals with an initial CR (183). These findings prompted the initiation of a pivotal, multicenter Phase II trial (NCT04268706) of the use of anti-CD30 CAR-T cells in patients with r/r HL; the trial is ongoing.

Grover et al. sought to enhance the effectiveness of anti-CD30 CAR-T cells in HL and CD30+ cutaneous T cell lymphoma by promoting migration to the TME through the expression of CC chemokine receptor 4. In a Phase I clinical trial (NCT03602157), all eight of the patients with HL and who underwent a disease assessment showed a response (six CRs and two PRs). Grover et al. indicated that five patients remained in remission, including one patient who still had a CR 2.5 years after treatment. With a median follow-up period of 12.7 months, the median PFS time for all 10 assessable patients was 5.2 months, while the median PFS time for HL patients only has not been determined (184). In a more recent clinical trial (NCT02690545), Voorhees et al. provided evidence of the remarkable efficacy of anti-CD30 CAR-T cells in 27 patients with r/r HL. Although a large proportion of the patients experienced a positive clinical response, a subset faced relapse and disease progression. After a median follow-up period of 9.5 months, 17 patients (63%) experienced disease progression (median PFS time: 352 days), and two (7%) succumbed to the disease (median OS time: not reached) (185).

#### 4.1.5 Multiple myeloma

In the KarMMa pivotal Phase II study, the use of idecabtagene vicleucel anti-BCMA CAR-T cell therapy led to frequent, good responses in patients with r/r MM and who had already been exposed to three lines of treatment. The ORR was 73%, and the CR rate was 33%. At a dose level of 450 × 10^6^ cells, the responses were more frequent and stronger: the ORR was 81%, and the CR rate was 39% (122).

Despite the observation of promising results for anti-BCMA CAR-T cell therapy in r/r MM, the long-term effectiveness was poor; a significant proportion of patients encountered disease relapse or progression (186, 187). The mechanisms of resistance are intricately linked to the interplay between anti-BCMA CAR-T cells, cancer cells, and the complex TME, which includes antigen evasion and the exhaustion of CAR-T cells (188). Fewer than 10% of adult patients with a recent diagnosis of MM will meet the FDA’s current eligibility criteria for fourth-line CAR-T cell therapy. Therefore, CAR-T cell treatment might be more effective if administered earlier in the course of the disease, when it is easier to reduce the tumor load (189). At present, a large number of promising target antigens for non-BCMA CAR-T cell therapies are being investigated. These include CD38, CD138, CD229, signaling lymphocytic activation molecule family member 7 (SLAMF7), a proliferation-inducing ligand, and G protein-coupled receptor, class C group 5 member D (GPRC5D) (190).

The combination of anti-BCMA CAR-T cell therapy with CD38 targeting has the potential to overcome the limitations observed with CARs that target the BCMA antigen only. In this context, Tang et al. developed bispecific CD38 and BCMA CAR-T cells (ChiCTR1900026286). The treatment gave an ORR of 87.5%, a 1-year PFS rate of 68.8%, and a manageable CRS incidence of 75%. However, it is important to note that even though the ORR was higher, anti-BCMA/CD38 CAR-T cells are not superior to anti-BCMA CAR-T cells because of the small number of treated patients and the absence of a head-to-head, randomized, controlled trial (191). By searching the ClinicalTrials.gov database, we identified six clinical trials of anti-CD38 CAR-T cells in management of MM (NCT03767751, NCT03473496, NCT03464916, NCT03271632, NCT06006741, and NCT05442580).

Concerning CD138, 5 patients with r/r MM have been treated with anti-CD138 CAR-T cells in a Phase I trial (NCT01886976) (192). By searching the ClinicalTrials.gov database, we identified seven clinical trials of anti-CD138 CAR-T cells for the treatment of MM (NCT03473496, NCT01886976, NCT03672318, NCT03271632, NCT03196414, NCT06006741, and NCT03778346). Another promising non-BCMA target for CAR-T cell therapy in the context of MM is the transmembrane receptor GPRC5D (193). An anti-GPRC5D CAR-T cell product has been studied initially in 18 patients, of whom 12 completed the treatment and 6 had previously received anti-BCMA CAR-T cells. The initial level of treatment efficacy was moderate, with two minimal responses, three PRs, three very good PRs, and two stringent CRs. Importantly, all patients who had previously received BCMA CAR-T cells responded to treatment. The expected adverse events were observed, with most of the patients experiencing grade 1-2 CRS (193). In a more recent Phase I dose-escalation trial conducted by Mailankody et al., GPRC5D-targeted CAR-T cell therapy was administered at four different dose levels to individuals with heavily pretreated MM. The ORR was 71% for the study population as a whole and 58% in patients having received doses ranging from 25 × 10^6^ to 150 × 10^6^ cells. Notably, some of the responding individuals had previously undergone anti-BCMA therapy (194).

It is noteworthy that other clinical trials are actively exploring alternative targets for CAR-T cell therapies in the context of r/r MM (190, 195).

### 4.2 CAR-NK cells in hematological cancers

Since the first clinical trial of CAR-NK cells recorded on ClinicalTrials.gov (NCT00995137, in 2009), 70 cancer-related studies of the safety, and efficacy of CAR-NK cells have been registered. The most recent trial results suggest that CAR-NK-cell therapies may be as efficacious as CAR-T cell therapies. However, it should be borne in mind that NK cells lack the long-term survival capacity of T lymphocytes. This major characteristic of NKs must be considered when evaluating the therapeutic potential of CAR-NK cells. Nevertheless, CAR-NK-based therapies have undergone extensive investigation for the treatment of hematological cancers. Preclinical studies have consistently demonstrated a significant advantage (both *in vitro* and *in vivo*) of CAR-expressing NK cells, relative to control NK cells. Research in this field has notably focused on CD19 - a pivotal area of interest following the FDA’s approval of anti-CD19 CAR-T cells (**Table 2**). Overall, a large body of compelling evidence shows that CAR-NK cells are highly efficacious in eliminating CD19-positive targets (65, 196, 197). The clinical trials mentioned below refer to data collected up to April 30^th^, 2024.

#### 4.2.1 B-cell lymphoblastic leukemia/lymphoma

Promising results have been obtained in a large number of preclinical and clinical studies of anti-CD19 CAR-NK cells for the treatment of B-cell malignancies. In a clinical trial (NCT03056339) conducted by Liu et al., 11 patients (five with CLL and six with NHL) were given a single dose of CAR-NK cells between June 2017 and February 2019. After monitoring for a median period of 13.8 months, eight (73%) of the 11 treated patients (four with CLL and four with NHL) exhibited an OR. Furthermore, 7 (64%) of the 11 patients achieved a CR (20).

The inherent heterogeneity of PB-NK cells and UCB-NK cells complicates the generation of standardized products. Hence, clinical settings have turned increasingly to homogeneous CAR-NK cells derived from iPSCs. One such product is FT596, a multiplexed iPSC-CAR-NK cell therapy engineered to incorporate a CD19-targeting CAR and IL-15/IL-15Rα fusion protein. This innovative therapy was evaluated in a Phase I clinical trial (NCT04245722) in 20 patients with r/r B-cell NHL or CLL and extensive prior treatments. FT596 was administered as a standalone therapy (10 patients) or in combination with rituximab (10 patients). Among the 17 patients assessed for efficacy after the initial FT596 treatment cycle, 5 out of 8 in the standalone treatment arm and 4 out of 9 in the combination treatment arm achieved a treatment response. When considering a single-dose level of ≥90 x10^6^ cells, 8 out of the 11 patients evaluated for efficacy achieved an OR, and 7 of these had a CR. Two of the four patients who had previously undergone CAR-T cell therapy and were treated with ≥90 x10^6^ cells achieved a CR (198). Ongoing clinical trials of anti-CD19 CAR-NK cells for the treatment of B-cell malignancies are summarized in **Table 3**.

**Table 3:** Ongoing clinical trials of anti-CD19 CAR-NK cells for the treatment of B-cell malignancies.

NCT number	Phase	Source	Signaling domain(s)	Gene expression	Type of cancer
NCT00995137	Phase I	NK-92 cells	4-1BB.CD3ζ	N/A	r/r B-ALL
NCT02892695	Phase I/II	NK-92 cells	CD28-4-1BB.CD3ζ	N/A	r/r B-cell cancers
NCT01974479	Phase I	Haploidentical NK cells	4-1BB.CD3ζ	N/A	r/r B-ALL
NCT03056339	Phase I/II	UCB	CD28.CD3ζ	IL-15	r/r B-cell cancers
NCT03824951	Early Phase I	iPSCs	N/A	N/A	r/r B-cell NHL
NCT03690310	Early Phase I	N/A	N/A	N/A	r/r B-cell lymphoma
NCT04245722	Phase I	iPSCs	NKG2D.2B4.CD3ζ	hnCD16 FcR.IL-15R	r/r B-cell lymphoma or CLL
NCT04639739	Early Phase I	N/A	N/A	N/A	r/r B-cell NHL
NCT04796675	Phase I	UCB	N/A	IL-15	r/r B-cell cancers
NCT04887012	Phase I	Haploidentical NK cells	N/A	N/A	r/r B-cell NHL
NCT05020678	Phase I	Allogeneic NK cells	OX40.CD3ζ	IL-15	r/r B-cell cancers
NCT05379647	Phase I	Allogeneic NK cells	N/A	N/A	r/r B-ALL
NCT05020015	Phase II	UCB	CD28.CD3ζ	IL-15	r/r B-cell NHL
NCT05410041	Phase I	PBMCs	N/A	N/A	r/r B-cell cancers
NCT05563545	Phase I	PBMCs	N/A	N/A	r/r CD19+AML
NCT04796688	Phase I	N/A	N/A	N/A	r/r B-cell cancers
NCT05472558	Phase I	UCB	N/A	N/A	r/r B-cell NHL
NCT05570188	Phase I/II	N/A	N/A	N/A	r/r B-cell cancers
NCT05645601	Phase I	Allogeneic NK cells	N/A	N/A	r/r B-cell cancers
NCT05654038	Phase I/II	N/A	N/A	N/A	r/r B-cell cancers
NCT05336409	Phase I	iPSCs	N/A	IL-15	r/r B-cell NHL
NCT05739227	Early Phase I	Allogeneic NK cells	N/A	N/A	r/r B-cell cancers
NCT05673447	Early Phase I	N/A	N/A	N/A	r/r DLBC

ALL, acute lymphocytic leukemia; AML, acute myeloid leukemia; CLL, chronic lymphocytic leukemia; DLBC, diffuse large B cell lymphoma; iPSC, induced pluripotent stem cells; PBMC, peripheral blood mononuclear cells; N/A, not available; NHL, Non-Hodgkin’s lymphoma; NK, natural killer; UCB, umbilical cord blood.

Dozens of ongoing clinical trials of CAR-NK cells are targeting various antigens in B-cell lymphoblastic leukemia or lymphoma. Results have not yet been published for most of the clinical trials listed. Given the lack of full datasets, a comprehensive analysis and comparison of these clinical trials is challenging. Nevertheless, it is noteworthy that the specific characteristics related to the CAR-NK cells might produce superior outcomes. As mentioned above, preclinical and clinical data have demonstrated that armoring CAR-NK cells with IL-15 results in enhanced NK cell activation, and cytotoxicity. Consequently, clinical trials of CAR-NK cells engineered to express the IL-15 or IL-15R genes (NCT03056339, NCT04245722, NCT05020015, and NCT05336409) are expected to report greater antitumor efficacy and prolonged persistence of genetically modified NK cells. In fact, as discussed above, Marin et al. reported highly encouraging findings from their initial phase I/II human trial involving UCB CD19 CAR-NK cells expressing sIL-15, and the inducible caspase-9 safety switch. The study included 37 patients with heavily treated r/r B cell malignancies. Single infusions of 1x10^5^, 1x10^6^ or 10x10^6^ CAR-NK cells per kg were not associated with the development of notable adverse events, such as CRS, neurotoxicity, or GvHD. The 1-year OS and PFS rates were 68% and 32%, respectively. Patients who achieved an OR had higher CAR-NK cell counts, and the latter persisted for longer (20, 67).

Moreover, the use of intracellular domains tailored to the NK cells’ distinctive functions and signaling pathways might yield better outcomes, relative to CAR-NK cells engineered with T-cell-specific intracellular domains. The NCT04245722 clinical trial appears to have given the most promising currently available data. The trial’s therapeutic cells are derived from iPSCs, which thereby provide an inexhaustible source of homogeneous NK cells and facilitate the development of a standardized, readily available treatment. The NK cells incorporate a cell-specific intracellular domain and have undergone additional genetic modifications, such as the expression of IL-15R (as mentioned above) and the high-affinity CD16 Fc receptor which enhances the cells’ ability to mediate ADCC against tumor targets. FT516 is potentially available for a larger number of patients, thanks to its off-the-shelf, mass production availability and facilitated multidose administration. Further research is required to confirm and extend these findings. In a Phase I trial in patients with r/r B-cell lymphoma, a combination of FT516 with rituximab was safe and well tolerated. Notably, FT516 administration yielded clinical responses (including some CRs) in heavily pretreated patients. Of the 11 patients treated with ≥90 x10^6^ FT516 cells, eight (72%) achieved an OR and seven a CR, including two patients whose disease had progressed after autologous anti-CD19 CAR-T cell therapy. However, two patients treated with the lowest dose of 30 x10^6^ FT516 cells experienced disease progression. Five of the eight responders remained in remission for between 4.6 and 9.5 months (199).

Other targets for CAR-NK cell therapy in B-cell malignancies have been tested in clinical trials; these include CD22, either alone (NCT03692767) or through a CD19/CD22 bispecific targeting (NCT03824964).

#### 4.2.2 T cell lymphoblastic leukemia/lymphoma

CAR-NK cells have demonstrated their efficacy in targeting various T cell TAAs, such as CD3, CD5, and CD7. Of the latter, CD5 has emerged as a primary focus of investigation for the treatment of T cell malignancies (200–202). Many Phase I and II clinical trials are presently assessing the safety, effectiveness, and ideal dose level of CAR-NK cells transduced with anti-CD5 (NCT05110742) or anti-CD7 (NCT02742727 and NCT04033302). The results of these studies have not yet been published.

#### 4.2.3 Acute myeloid leukemia

Studies demonstrating the effective internalization of antibodies binding to CD33 have opened up new perspective for treating CD33-bearing cancers (203, 204). Tang et al. assessed the safety (NCT02944162) of anti-CD33 CAR-NK-92 cells as a treatment for three patients with r/r AML. Although the results of this Phase I trial did not reveal significant clinical effectiveness, they did show that the therapy could be safely administered to patients with a high tumor burden (205). There are five ongoing clinical trials of anti-CD33 CAR-NK cells in AML, two of which feature cells targeting both CD33 and CLL-1. Other targets (such as CD70 and CD123) are also under clinical investigation (**Table 4**).

**Table 4:** Ongoing clinical trials of CAR-NK cells in AML.

NCT number	Phase	CAR	Source	Signaling domains	Gene expression
NCT02944162	Phase I/II	CD33	NK-92 cells	CD28.4-1BB.CD3ζ	N/A
NCT05008575	Phase I	CD33	N/A	N/A	N/A
NCT05665075	Phase I	CD33	iPSCs	N/A	hnCD16 FcR.IL-15R
NCT05601466	Phase I	CD33	iPSCs	N/A	hnCD16 FcR.IL-15R
NCT05215015	Early Phase I	CD33 and CLL-1	N/A	N/A	N/A
NCT05987696	Phase I	CD33/CLL-1	iPSCs	N/A	N/A
NCT06367673	Phase I	CD33 or CLL-1	iPSCs	N/A	N/A
NCT06325748	Phase I	CD33 and/or FLT3	Allogeneic NK cells	N/A	N/A
NCT06027853	Phase I	CLL-1	iPSCs	N/A	N/A
NCT06307054	Phase I	CLL-1	N/A	N/A	N/A
NCT05092451	Phase I/II	CD70	UCB	N/A	IL-15
NCT06201247	Early Phase I	CD123	N/A	N/A	N/A
NCT05574608	Early Phase I	CD123	Allogeneic NK cells	N/A	N/A
NCT06006403	Phase I/II	CD123	N/A	N/A	N/A
NCT04623944	Phase I	NKG2D	Allogeneic NK cells	N/A	N/A

CAR, chimeric antigen receptor; iPSC, induced pluripotent stem cells; N/A, not available; NK, natural killer; NKG2D, natural killer group 2member D; UCB, umbilical cord blood.

#### 4.2.4 Hodgkin lymphoma

To date, CAR-NK cells have not been tested clinically for the treatment of HL.

#### 4.2.5 Multiple myeloma

As seen with CAR-T cells, BCMA is a promising target for CAR-NK cell-based therapies for MM. A Phase I trial of the efficacy and safety of anti-BCMA CAR-NK cells in MM therapy is currently being conducted by Dhakal et al. None of the nine patients having received CAR-NK cell infusions (three of whom also received anti-CD38 daratumumab as part of combination therapy) developed CRS or ICANS. Encouragingly, a patient having received monotherapy with 300x10^6^ anti-BCMA CAR-NK cells achieved a PR, as did two patients having 100x10^6^ cells of anti-BCMA CAR-NK cells plus daratumumab (206). At present, these are the only published preliminary data on CAR-NK cells targeting BCMA, and so it is hard to predict any future specific outcomes on this basis. Three other clinical trials of anti-BCMA CAR-NK cells in the treatment of MM are currently underway (**Table 5**). Other targets have also shown positive results in preclinical studies [such as: SLAMF7 (207), CD138 (208), and CD38 (209, 210)] and might soon move into clinical development.

**Table 5:** Ongoing clinical trials of anti-BCMA CAR-NK cells in the treatment of MM.

NCT number	Phase	CAR	Source	Signaling domains	Gene expression
NCT05652530	Early Phase I	BCMA	N/A	N/A	N/A
NCT05008536	Early Phase I	BCMA	UCB	N/A	N/A
NCT03940833	Phase I/II	BCMA	NK-92 cells	N/A	N/A
NCT05182073	Phase I	BCMA	iPSCs	N/A	hnCD16 FcR.IL-15R
NCT06045091	Early Phase I	BCMA	N/A	N/A	N/A
NCT06242249	Phase I/II	BCMA	N/A	N/A	N/A

BCMA, B-cell maturation antigen; CAR, chimeric antigen receptor; iPSC, induced pluripotent stem cells; N/A, not available; NK, natural killer; UCB, umbilical cord blood.

### 4.3 Clinical results synthesis

In the clinical aspect of hematological cancers, CD19 CAR-T cells have been transformative in the treatment of B-cell ALL, NHL, and most recently MM (211) (**Table 2**). Overall, the data have demonstrated that CD19-targeted CAR-T cells can achieve extended remissions in patients with B cell malignancies, often with minimal long-term toxicities, and may even be curative for some. CR rates were observed at 40-54% for aggressive B cell lymphomas, 67% for mantle cell lymphoma, and 69-74% for indolent B cell lymphomas (119, 121, 212). These impressive results marked a major shift in treatment for these patients, leading to FDA approval for these conditions. Additionally, CD19-targeted CAR-T cells have achieved CR rates of 71-81% in patients with r/r B-ALL, who have limited treatment options, also resulting in FDA approvals (118, 213). In more recent developments, CAR-T cells targeting BCMA have achieved overall response rates of 73-98% in patients with r/r MM, adding new FDA approvals for this indication as well (214, 215). However, as this therapeutic strategy is expanded to other hematological malignancies, new challenges have emerged. For instance, targeting antigens like CD33 or CD123 for AML and CD5 or CD7 for T-ALL poses the risk of off-target side effects (216, 217). In addition, despite the therapy’s remarkable efficacy, a major challenge observed in 30%-70% of patients with recurrent disease is antigen loss or downregulation (218). In patients with ALL undergoing CAR-T cell therapy, relapse rates range from 10% to 57%. Those who experience antigen loss often face more limited treatment options (219, 220). Thus, tumor escape through antigen loss, particularly with CD19, has highlighted the need for alternative options. Various strategies are currently being investigated to address this issue and enhance treatment efficacy, such as dual-targeted CAR-T cells, innovative combinatorial approaches involving CAR-T cell therapy and other immunotherapeutic agents (mainly immune checkpoint inhibitors, bispecific antibodies, oncolytic viruses, small molecule inhibitors or antibody-drug conjugates), or logic-gated CAR-T cells (218).

In contrast, CD19 CAR-NK cells offer a different therapeutic profile. Although newer in clinical trials, they show promise due to their inherent ability to kill cancer cells without pre-sensitization, lower toxicity risk, and the potential for allogeneic use (221). Since the first clinical trial involving CD33 CAR-NK-92 cells in patients with r/r AML, the field has rapidly evolved (205). Rezvani’s team conducted the first clinical trial involving CD19-targeted CAR UCB-NK cells in 37 patients with r/r CD19-positive malignancies (NCT03056339). The 1-year OS and PFS rates were 68% and 32%, respectively (20, 67). Another promising development in this area is represented by the iPSC-derived off-the-shelf CD19-directed CAR-NK cell product (FT596) with 8 of the 11 evaluable patients displaying an OR, 7 of them achieving CR (222). These findings highlight the potential of CD19 CAR-NK cells as a safer and potentially more scalable alternative to CAR-T cell therapy, although the comparison isn’t entirely equitable given the different stages of clinical trials. As clinical trials continue to evolve, the roles of CD19 CAR-T and CAR-NK cells are becoming increasingly defined, with each offering unique benefits that could complement the other in the quest to improve outcomes for patients with r/r hematological cancers.

For other tumor antigens, no true comparison can be made due to the lack of robust clinical data for CAR-NK cell- based trials.

## 5 Futures directions

CAR-T cell-based immunotherapies have revolutionized the treatment of hematological cancers over the past decade. CAR-T cells have shown significant efficiency, achieving strong and durable responses in clinical trials. CAR-NK cells have provided advantages such as reduced CRS, and the potential for off-the-shelf allogeneic therapies. However, they have faced challenges like limited cell persistence and less efficient tumor targeting. In order to address the short lifespan of ACT cells, the latter have been engineered to produce cytokines that enhance their proliferation and persistence *in vivo*. Another strategy to improve the persistence of immune cell-based therapies is the infusion of memory cells with enhanced proliferative and survival capacities and potent antitumor functions. CAR-T cells with long-lived memory phenotypes, such as stem cell memory T cells and central memory T cells, have been well-documented for their origins from adaptive immune T cells and are correlated with durable remissions in patients with hematological malignancies (223–225). However, as the identification of memory NK (mNK) populations is relatively recent, integrating the characteristics of these populations represents a novel approach to enhancing the persistence of CAR-NK therapies. Recent studies have shown that cytokine-induced memory-like (CIML) CAR-NK cells exhibit superior anti-tumor activity compared to conventional CAR-NK cells. This improvement is attributed to the memory-like properties conferred by prior exposure to specific cytokines (notably IL-12, IL-15, and IL-18), which enhance the CAR-NK cells’ survival, proliferation, and cytotoxicity *in vivo* (226–228).

For instance, CIML CAR-NK cells targeting CD19 in B-cell malignancies have shown prolonged survival and reduced tumor progression in preclinical models, highlighting the potential of this approach to improve outcomes for patients with refractory cancers. Additionally, the ability of CIML CAR-NK cells to maintain high levels of IFN-γ production and ADCC further supports their utility in combination therapies with monoclonal antibodies (196, 227, 229). In this respect, it is important to highlight the promising clinical applications of FcϵRIγ-deficient NK cells (referred to as g-NK cells), which represent a cutting-edge advancement in NK cell-based immunotherapy. This newly discovered subset of human NK cells, characterized by the absence of the FcϵRIγ adapter protein, has demonstrated a multi-fold increase in ADCC activity following CD16 crosslinking (230). A first-in-human Phase I clinical trial is currently underway to evaluate the safety and efficacy of g-NK cells in patients with r/r NHL and MM. This trial is particularly significant as it explores the potential of g-NK cells, in combination with rituximab for NHL and daratumumab for MM, to address the limitations of current NK cell therapies, such as limited durability and potency (NCT05012345). Furthermore, the versatility of g-NK cells suggests potential applications beyond hematological cancers. Their ability to be combined with a wide range of therapeutic antibodies opens up possibilities for treating solid tumors.

Advances in nanocarriers/LNP platforms, CRISPR-Cas9, and transposon-based technologies are enhancing the safety, cost-effectiveness, and precision of these therapies. Research continues to refine these approaches to improve the generation and efficacy of both CAR-T and CAR-NK cells.

Although CAR-engineered immune cells hold considerable promise for treating hematological cancers, applying this approach to solid tumors presents several challenges (231, 232). Over the past decades, numerous target antigens for solid tumors have been identified, with some demonstrating safety and feasibility in preclinical and clinical studies. Despite ongoing research, no CAR-T cell therapies have been approved for clinical use yet (233), but the first TCR gene therapy, named Tecelra, has just been approved by the FDA (August 2, 2024). This innovative treatment utilizes the patient’s own T cells, which are modified to express a TCR targeting melanoma-associated antigen A4 (MAGE-A4), a protein present on synovial sarcoma cells (234).

In light of the limited success of CAR-T cell therapies for solid tumors, recent research has turned to CAR-NK cells as a potential alternative and has explored their efficacy against various solid tumor antigens, including HER-2 (235) and CD73 (236) in lung cancer, EGFRvIII in glioblastoma (237), EGFR in triple-negative breast cancer (238), mesothelin in gastric cancer (239), c−MET in liver cancer (240), PSMA in prostate cancer (241), EpCAM in colorectal cancer (242), and GPC3 in HCC (33). These studies yielded promising results.

These non-exhaustive preclinical studies underlined the importance of CAR-NK cells in the elimination of solid tumors, and paved the way for the development of clinical trials. Currently, more than twenty Phase I clinical trials are underway, exploring various CAR-NK strategies across multiple types of solid cancers (ClinicalTrials.gov). The expected results from these trials will allow for a more thorough evaluation of the efficacy of CAR-NK cells in treating solid tumors.

Novel concepts are constantly being developed to enhance therapeutic efficacy of ACT. In this regard, comparing CARs and TCRs is complex due to the diversity in CAR structures and ligands. However, the HLA-independent TCR (HIT) engineering designed by Michel Sadelain shows promising results since even both CARs and HIT receptors effectively kill target cells, HIT receptors have been shown to be more sensitive, especially when target antigens are fewer. *In vitro* experiments suggest that HIT receptors are at least ten times more sensitive than CARs, which may explain their superior ability to eliminate tumors compared to CAR-T cells (243).

In summary, ACT is becoming a clinical reality and might revolutionize precision immunotherapy. This approach should bring new hope to patients with difficult-to-treat cancers, including solid tumors. CAR-T cells and CAR-NK cells are promising therapeutics and are taking clinicians into a future in which cancer treatment is always personalized. The results of the many ongoing clinical trials are likely to provide comprehensive insights into the safety and efficacy of CAR-T cells and CAR-NK cells.
